# Comparative analysis of fluoride release from type II GIC, ketac molar and ketac universal: An *in vitro* study

**DOI:** 10.6026/973206300223390

**Published:** 2026-06-30

**Authors:** Sushmita Shivanna, Prakash H, Juan M, Murali Sivakumar, Megavarnan Ravi

**Affiliations:** 1 Department of Conservative Dentistry and Endodontics, Sathyabama Dental College & Hospital, Tamil Nadu, India

**Keywords:** Fluoride release, GIC cements, caries prevention, high-viscosity GIC, ketac molar, ketac universal, Type II GIC, UV spectrophotometer

## Abstract

Limited evidence exists regarding the comparative fluoride release of different glass ionomer restorative materials used in clinical dentistry. Therefore, it is of interest to evaluate and compare the fluoride release of Type II GIC, Ketac Molar, and Ketac Universal at different time intervals. Fluoride ion release from these materials was assessed over 1, 7, and 28 days using a UV-visible spectrophotometer. Significant differences in fluoride release were observed among the materials (p < 0.001). Ketac Universal showed the highest fluoride release at all evaluated intervals. Fluoride release from Ketac Molar and Ketac Universal increased over time, whereas Type II GIC remained relatively unchanged. The superior fluoride release of Ketac Universal suggests its potential clinical advantage for long-term caries prevention in patients at high risk of dental caries.

## Background:

Fluoride-releasing restorative materials are essential in the prevention and control of dental caries, particularly in conservative and preventive dentistry. The release of fluoride ions facilitates the remineralization of tooth structures and produces a cariostatic effect by inhibiting acid production [1, 2]. One such material is Glass ionomer cements (GIC's), widely used for its biocompatibility, chemical bonding properties and biphasic fluoride release, characterized by an initial burst within the first 24-48 hours, followed by a sustained low-level release over time [1, 2]. This pattern of fluoride release is particularly advantageous for individuals at high risk of dental caries, as it fosters a fluoride-rich environment that promotes tooth remineralization and enhances resistance to caries [3]. High-viscosity Type II GIC's such as Ketac Molar and Ketac Universal are frequently used in minimally invasive dentistry, atraumatic restorative treatment (ART), and school-based preventive programs [4, 5]. Ketac Molar is known for its fluoride durability, while Ketac Universal, offers improved handling and does not require surface conditioning [6]. Both materials demonstrate prolonged fluoride release underscoring their potential long-term benefits [7, 8]. Clinically, these materials are particularly suitable for use in primary teeth due to their ease of placement, fluoride release, and reduced need for extensive cavity preparation [9]. Their fluoride releasing capability is assessed using UV Spectrophotometer. Although several *in vitro* studies have evaluated the fluoride-releasing capacity of various GICs [10, 11] comparative study with Ketac Molar, Ketac Universal, and conventional Type II GIC analysing their fluoride release in a standardized environment has not been done. Therefore, it is of interest to report and compare the release of fluoride ions from Type II GIC, Ketac Universal and Ketac Molar at intervals of 1, 7, and 28 days.

## Materials and Methods:

## Study design:

This study is an *in vitro* comparative study.

## Sample size estimation:

The sample size was calculated a priori using G Power (v3.1.9.4) software. Parameters were set at a significance level (α) of 0.06, power of the study 0.85, effect size-0.5. An ANOVA, fixed-effects, omnibus, one-way model was used. A total sample size of 45 (n=45) was calculated with each group including 15 samples.

## Materials used:

[1] Type II GIC (GC Corporation, Japan) (Control)

[2] Ketac Molar (3M ESPE, Germany)

[3] Ketac Universal (3M ESPE, Germany)

[4] Distilled water

[5] Stainless steel mould

[6] Glass Slab

[7] Mylar strips

[8] Polypropylene containers

[9] UV-visible spectrophotometer (JASCO corporation (Japan), Model: JASCO V-730)

## Sample preparation and Fluoride measurement:

In this study, forty-five pellets (n=15 per group) of Type II GIC cement, Ketac Molar, and Ketac Universal were made using stainless steel moulds measuring 10 mm in diameter and 2 mm in thickness. The cement was mixed following the manufacturer's guidelines, and the resulting material was promptly covered with a Mylar strip, then sandwiched between glass slabs and excess material were removed (Figure 1A - see PDF). Once set, any excess material around the edges was trimmed with a scalpel, and the pellets were carefully removed from the moulds (Figure 1B, Figure 1C, Figure 1D - see PDF). Samples that exhibited voids or uneven surfaces were not included in the study. Group 1 consisted of Type II GIC, Group 2 of Ketac Molar, and Group 3 of Ketac Universal. Each group was further divided into three subgroups, each containing five samples placed in polypropylene containers and immersed in 100 ml of distilled water (pH-7) within an incubator set at 37°C (Figure 2E, Figure 2F, Figure 2G - see PDF). Fluoride release was assessed on the 1st, 7th, and 28th days. The time intervals of 24 hours, 7 days, and 28 days were chosen to represent the initial burst release, early sustained release, and medium- term fluoride availability. Fluoride ion concentrations were quantified using a standard calibration curve. Absorbance was measured at 570 nm using a UV-visible spectrophotometer (JASCO V-730, JASCO Corporation, Japan) (Figure 2H - see PDF), and a standard curve was plotted. The fluoride concentration in each sample was then determined by comparing its absorbance to the calibration curve (Figure 2I, Figure 2J - see PDF).

## Statistical analysis:

Data were analysed using SPSS (IBM) via one-way ANOVA and Tukey's post hoc test. A p-value <0.05 was considered statistically significant.

## Results:

This study evaluated the fluoride release from three GIC based materials- Type II GIC, Ketac Molar, and Ketac Universal-on Days 1, 7, and 28. Ketac universal consistently showed the highest mean fluoride release value at all points followed by Ketac molar and Type II GIC. On the first day, the mean fluoride release values (Mean ± SD) were 0.040 ± 0.005 ppm for Type II GIC, 0.051 ± 0.008 ppm for Ketac Molar, and 0.061 ± 0.002 ppm for Ketac Universal. By Day 7, these values increased to 0.065 ± 0.012 ppm, 0.118 ± 0.029 ppm, and 0.210 ± 0.022 ppm, respectively. On Day 28, the fluoride release reached 0.090 ± 0.007 ppm for Type II GIC, 0.149 ± 0.026 ppm for Ketac Molar, and 0.286 ± 0.025 ppm for Ketac Universal (Figure 3 - see PDF). One way ANOVA on intergroup analysis showed a statistically significant differences in fluoride release among all three materials at each time points (Day 1, 7, 28: p<0.001). Tukey's post hoc revealed that Ketac universal released significantly more fluoride compared to Ketac molar (Day 1: p=0.018, Day 7,28: p<0.001) and type II GIC (Day 1,7,28: p<0.001). Ketac molar recorded a more fluoride release compared to Type II GIC (Day 1: p<0.001, Day 7: p=0.007 and Day 28: p=0.002) (Table 1, Table 2). Intragroup analysis using One way ANOVA indicated that Type II GIC did not show a statistically significant fluoride release over all three-time intervals (p=0.243). In contrast, Ketac molar and Ketac universal recorded significantly high fluoride release over time (p<0.001). Tukey's post hoc analysis showed that these two materials had statistically significant fluoride release over the time interval indicating sustained and progressive fluoride releasing profile (Table 3, Table 4).

## Discussion:

Glass ionomer cements (GICs) are conventional cements known for their ability to chemically bond to the tooth structure and release fluoride, a crucial feature that enhances their potential to prevent cavities [12]. This study evaluated the fluoride release patterns of three GIC-based restorative materials-Type II GIC (Conventional), Ketac Molar, and Ketac Universal-on days 1, 7, and 28 respectively, following the typical biphasic pattern [2] of fluoride release characterized by an initial "burst release" because of explosion effect, [13] succeeded by a diffusion-driven release phase and provides sustained low level fluoride release and followed by long-term sustained release phase [14]. Ketac Molar, a high-viscosity GIC, is designed with a higher powder-to-liquid ratio and a dense matrix, which enhances mechanical strength while still allowing fluoride ion release. This formulation balances physical durability with ion exchange properties, making it a dependable choice for stress-bearing areas with moderate fluoride delivery. Ketac Universal showed the highest fluoride release at all measured intervals. This could be due to its refined composition, which includes finely ground fluor aluminosilicate glass, polyacrylic acid, and tartaric acid [15]. The smaller particle size increases the reactive surface area, enhancing ion mobility and enabling sustained fluoride release over time. In our study Ketac Universal demonstrated a notable initial release on the first day, attributed to the "Superficial rinsing effect." This effect arises when water or another medium interacts with the material's surface. Subsequently, there was a gradual reduction in release observed on days 7 and 28, yet it continued to achieve the highest overall release compared to other two materials. However, Ketac Molar showed a significant initial release with a moderate decrease over time, still outperforming Type II GIC. This superiority of Ketac molar over type II GIC may be attributed to finely controlled micronization of GIC particles providing a larger surface area leading to increased acid base reactivity and hence increased capacity of fluoride release compared to the latter [16]. In contrast, Type II GIC had the lowest release at all intervals, reflecting its older formulation and limited surface reactivity [17]. Group 3 (Ketac Universal) outperformed Group 2 (Ketac Molar) at all intervals, due to its enhanced glass particle fineness and matrix composition that promotes fluoride diffusion. There was superior performance of Group 3 over Group 1, which aligns with previous findings highlighting the importance of modern formulation advancements for sustained fluoride availability [18]. However, Group 2 demonstrated better fluoride release than Type II GIC, attributed to its high-viscosity formulation and increased reactivity, supporting its use in high caries-risk patients [13]. Moreover, Group 3 emerged as the most effective fluoride-releasing material among the three, due to its optimized formulation, smaller particle size, and enhanced ion mobility. Its ability to maintain high fluoride levels over time supports its application in patients requiring long-term cariostatic protection. Group 2, while not as efficient in fluoride release as Group 3, still demonstrated superior performance compared to Group 1. Its formulation strikes a balance between mechanical resilience and therapeutic fluoride delivery. Whereas, in Group 1, though widely used and cost-effective, lags behind in fluoride release capabilities. Its conventional formulation limits its efficacy in high caries-risk environments.

## Conclusion:

We show that Ketac Universal had notably higher fluoride levels than both Ketac Molar and traditional Type II GIC at every time point assessed. Its steady and prolonged fluoride release pattern highlights its potential efficacy in promoting long-term caries prevention. These data shows for the preferential application of Ketac Universal in clinical situations requiring prolonged fluoride presence, especially among populations at high risk for caries and in preventive dental initiatives. Additional *in vivo* research is suggested to confirm these findings in the dynamic environment of the oral cavity.

## Figures and Tables

**Table 1:** One-Way ANOVA comparing fluoride release between materials at each time point

**Time Point**	**Sum of Squares**	**df**	**Mean Square**	**F**	**Sig. (p-value)**
Day 1	0.001	2	0.001	18.649	<0.001*
Day 7	0.054	2	0.027	54.372	<0.001*
Day 28	0.101	2	0.051	115.713	<0.001*
One way ANOVA analysis,
* Statistically
Significant p < 0.05

**Table 2:** Tukey HSD post hoc comparison of fluoride release between materials

**Time**	**Comparison**	**Mean Difference **	**Std. Error**	**Sig.**	**95% CI Lower Bound**	**95% CI Upper Bound**
Day 1	Type II GIC vs Ketac Molar	-0.00998	0.0035	0.036*	-0.01932	-0.00064
	Type II GIC vs Ketac Universal	-0.02136	0.0035	<0.001*	-0.0307	-0.01202
	Ketac Molar vs Ketac Universal	-0.01138	0.0035	0.018*	-0.02072	-0.00204
Day 7	Type II GIC vs Ketac Molar	-0.00692	0.0035	0.007	-0.01626	-0.00242
	Type II GIC vs Ketac Universal	-0.09868	0.0035	<0.001*	-0.10802	-0.08934
	Ketac Molar vs Ketac Universal	-0.09176	0.0035	<0.001*	-0.1011	-0.08242
Day 28	Type II GIC vs Ketac Molar	-0.00128	0.0035	0.002*	-0.01062	0.00806
	Type II GIC vs Ketac Universal	-0.14128	0.0035	<0.001*	-0.15062	-0.13194
	Ketac Molar vs Ketac Universal	-0.14	0.0035	<0.001*	-0.14934	-0.13066
Tukey's Post Hoc analysis,
* Statistically
Significant p < 0.05

**Table 3:** One-Way ANOVA comparing fluoride release over time within each material

**Material**	**Sum of Squares**	**df**	**Mean Square**	**F**	**Sig. (p-value)**
Type II GIC	0.001	2	0	1.595	0.243
Ketac Molar	0.006	2	0.003	47.449	<0.001*
Ketac Universal	0.094	2	0.047	34.82	<0.001*
One way ANOVA analysis,
* Statistically
Significant p < 0.05

**Table 4:** Tukey HSD post-hoc comparison of fluoride release over time within each material

**Material**	**Comparison**	**Mean Difference**	**Std. Error**	**Sig.**	**95% CI Lower Bound**	**95% CI Upper Bound**
Ketac Molar	Day 1 vs Day 7	-0.0256	0.0051	0.001*	-0.03934	-0.01186
	Day 1 vs Day 28	-0.0502	0.0051	<0.001*	-0.06394	-0.03646
	Day 7 vs Day 28	-0.0246	0.0051	0.001*	-0.03834	-0.01086
Ketac Universal	Day 1 vs Day 7	-0.1627	0.0132	<0.001*	-0.2083	-0.1171
	Day 1 vs Day 28	-0.2213	0.0132	<0.001*	-0.2669	-0.1757
	Day 7 vs Day 28	-0.0586	0.0132	0.004*	-0.1042	-0.01300*
Tukey's Post Hoc analysis,
* Statistically
Significant p < 0.05
